# WISP1 alleviates lipid deposition in macrophages via the PPARγ/CD36 pathway in the plaque formation of atherosclerosis

**DOI:** 10.1111/jcmm.15783

**Published:** 2020-08-27

**Authors:** Dian Liu, Xuyang Wang, Mingjun Zhang, Jingjing Tian, Ming Liu, Tao Jin, Jinyu Pan, Mingxiao Gao, Fengshuang An

**Affiliations:** ^1^ The Key Laboratory of Cardiovascular Remodeling and Function Research Chinese Ministry of Education Chinese National Health Commission and Chinese Academy of Medical Sciences The State and Shandong Province Joint Key Laboratory of Translational Cardiovascular Medicine Department of Cardiology Qilu Hospital Cheeloo College of Medicine Shandong University Jinan China; ^2^ Department of Cardiology Shandong Provincial The First Affiliated Hospital of Shandong First Medical University Jinan China; ^3^ ICMA CENTER University of Reading Reading UK

**Keywords:** atherosclerosis, CD36, lipid deposition, macrophage, PPRAγ, SR‐A, WISP1

## Abstract

Lipid deposition in macrophages plays an important role in atherosclerosis. The WNT1‐inducible signalling pathway protein 1(WISP1) can promote proliferation and migration of smooth muscle cells. Its expression is up‐regulated in obesity, which is associated with atherosclerosis, but the effect of WISP1 on atherosclerosis remains unclear. Thus, the objective of our study was to elucidate the role of WISP and its mechanism of action in atherosclerosis via in vivo and in vitro experiments. In our experiment, ApoE‐/‐ mice were divided into 5 groups: control, high‐fat diet (HFD), null lentivirus (HFD + NC), lentivirus WISP1 (HFD + IvWISP1) and WISP1‐shRNA (HFD + shWISP1). Oil Red O staining, immunofluorescence and immunohistochemistry of the aortic sinuses were conducted. Macrophages (RAW264.7 cell lines and peritoneal macrophages) were stimulated with 50 μg/mL oxidized low‐density lipoprotein (ox‐LDL); then, the reactive oxygen species (ROS) level was measured. Oil Red O staining and Dil‐ox‐LDL (ox‐LDL with Dil dye) uptake measurements were used to test lipid deposition of peritoneal macrophages. WISP1, CD36, SR‐A and PPARγ expression levels were measured via Western blotting and ELISA. The results showed that HFD mice had increased WISP1, CD36 and SR‐A levels. The plaque lesion area increased when WISP1 was down‐regulated, and lipid uptake and foam cell formation were inhibited when WISP1 was up‐regulated. Treatment of RAW264.7 cell lines with ox‐LDL increased WISP1 expression via activation of the Wnt5a/β‐catenin pathway, whereas ROS inhibition reduced WISP1 expression. Moreover, WISP1 down‐regulated CD36 and SR‐A expression, and Oil Red O staining and Dil‐ox‐LDL uptake measurement showed that WISP1 down‐regulated lipid deposition in macrophages. These results clearly demonstrate that WISP1 is activated by ox‐LDL at high ROS levels and can alleviate lipid deposition in atherosclerosis through the PPARγ/CD36 pathway.

## 1 INTRODUCTION

Atherosclerosis is the leading cause of cardiovascular disease. It is a complex process that includes endothelial dysfunction, lipid deposition, and proliferation and migration of smooth muscle cells.^1^, ^2^, ^3^ Formation of macrophage foam cells is characteristic of early atherosclerotic lesions, ^2^ and oxidized low‐density lipoprotein (ox‐LDL) is absorbed by macrophages with the help of scavenger receptors (SRs). ^4^ Ox‐LDL, lysophosphatidylcholine and oxidized fatty acids induce the expression of these scavenger receptors, such as CD36, SR class A (SR‐A) and LOX‐1.^5^ Thus, peroxisome proliferator‐activated receptor γ (PPARγ), a ligand‐activated transcription factor known to regulate fatty acid metabolism, plays a role in lipid deposition.^6^ After its activation, PPARγ gene transcription is regulated to modify lipid metabolism, and it has important effects on cell proliferation, differentiation and inflammatory responses.^7^, ^8^, ^9^ Additionally, studies have found that PPARγ is the main factor regulating the uptake of ox‐LDL by CD36, which affects the generation of foam cells by influencing lipid metabolism in macrophages.^10^, ^11^, ^12^ CD36 belongs to the class B scavenger receptor family, which is localized in various cell types, such as monocytes/macrophages, adipocytes, endothelial and smooth muscle cells.^13^, ^14^, ^15^, ^16^ Ox‐LDL, apoptotic cells and advanced glycation end products (AGEs) are the most noticeable substances involved in atherosclerosis development that interact with CD36.^17^ Furthermore, CD36 and ox‐LDL binding can activate signalling pathways, including those of protein kinases that lead to the development of atherosclerosis.^18^, ^19^, ^20^ Additionally, it is reported that the knockout of SR‐A, a kind of cell surface glycoprotein that belongs to the SR family,^21^ can accelerate atherosclerosis.^22^

The WNT signalling pathway is rather a conservative pathway in biological evolution that is closely related to embryo formation, development and cell differentiation. In recent years, studies on the role of the WNT pathway in cardiovascular diseases have attracted much attention, as it participates in the adhesion between endothelium and monocytes, regulates the function of smooth muscle cells, promotes vascular calcification^23^, ^24^ and plays an important role in the adipogenic differentiation of cells.^25^ WNT‐inducible signalling pathway protein 1 (WISP1) belongs to the CCN family of extracellular matrix proteins, and it is identified as a downstream target gene of the canonical WNT signalling pathway.^8^ WISP1 plays an important role in the inflammatory process, and its expression is closely related to the severity of osteoarthritis due to its effect on the expression of chondrocytes, macrophage matrix metalloproteinases and proteoglycan enzymes.^26^ Further, WISP1 expression was found to increase significantly in neurons under oxidative stress and fracture repair.^27^ Studies have proven that WISP1 can accelerate the migration and proliferation of smooth muscle cells to thicken the intima of blood vessels.^28^ Other studies have shown that obesity, which is associated with atherosclerosis, leads to the up‐regulation of WISP1.^29^ However, whether WISP1 plays a role in the lipid deposition in atherosclerosis remains unclear.

In this study, we investigated the relationship between WISP1 and atherosclerosis, the function of WISP1 during atherosclerotic plaque formation and progression, and the specific role(s) and molecular mechanism(s) of action of WISP1. The results of our in vitro and in vivo experiments point to a possible mechanism of WISP1 in the progression of atherosclerosis.

## 2 METHODS

### 2.1 Animals

All mouse studies were sanctioned by the Animal Ethics Committee of Shandong University; the care and use of animals followed the guidelines on animal ethics. Male ApoE‐/‐ mice (8 weeks old, n = 100) were obtained from HFK Bioscience Company (Beijing, China). All the ApoE‐/‐ mice were randomly divided into 5 groups (n = 20 per group): Group 1: control group; Group 2: high‐fat diet (HFD); Group 3: HFD + NC (null lentivirus, 5’‐ GTTCTCCGAACGTGTCACGT −3’); Group 4: HFD + IvWISP1 (lentivirus WISP1); and Group 5: HFD + shWISP1 (WISP1‐shRNA, 5’‐CCA CTA GAG GAA ACG ACT A‐3’) (GenePharma, China). Group 1 mice were fed with a diet of 5% fat without cholesterol, whereas the other groups were fed with an HFD (16% fat and 0.25% cholesterol). All mice were provided with food and water and subjected to a light‐dark cycle in an environment at 20℃‐22℃ and 50%‐60% humidity. After 7 days of feeding to adapt to the environment, the mice were injected with null lentivirus (Group 3), shWISP1 (Group 5) or lentivirus WISP1 (Group 4) via the caudal vein with a total lentivector dose of 2 × 10^7^ TU/mouse. After 12 weeks, the mice were anaesthetized intraperitoneally with 3% pentobarbital sodium (40 mg/kg) and killed. The hearts were gathered for histological staining, the aortas were gathered for Western blotting and Oil Red O staining, and the serum samples were collected for blood lipid detection and ELISA. The effectiveness of the lentivirus was confirmed by the expression of WISP1 in the serum and tissues of the mice injected with lentivirus.

### 2.2 Cell culture

In this study, macrophages (peritoneal macrophages and RAW264.7 cell lines) were cultured in the Dulbecco's modified eagle medium (DMEM glucose 5.5 mM) supplemented with 10% foetal bovine serum (FBS) in an atmosphere at 37℃ and 5% CO2. Peritoneal macrophages were obtained from male C57BL/6 mice (6 weeks old), and all cells were plated in 6‐well dishes. In the reactive oxygen species (ROS)‐related experiment, 5 mmol/L N‐acetylcysteine (NAC, Beyotime, Beijing, China) served as an ROS inhibitor under the condition of ox‐LDL (Yiyuan Biotechnology, China) stimulation for 24 h. Null lentivirus (MOI 100, 5’‐ GTTCTCCGAACGTGTCACGT −3’), shWISP1 (MOI 100, 5’‐CCA CTA GAG GAA ACG ACT A‐3’) and lentivirus WISP1 (MOI 120) (GenePharma, China) were transfected into RAW264.7 cell lines and peritoneal macrophages to overexpress and inhibit WISP1. The efficiency of the lentivirus transfection was reflected by WISP1 expression in transfected cells.

Cells were cultured for 24 h and then cultured for another 24 h after replacing the culture medium. Protein was extracted from the cells after 50 μg/mL ox‐LDL was added into the medium for 12 h. PPARγ signalling was blocked via a 2‐h treatment with PPARγ inhibitor T0070907 (20 μmol/L) (Selleck, America). RAW264.7 cells were transfected with negative control siRNA or WISP1 siRNA, which had WISP1 knockdown performed via Lipofectamine 2000 treatment for 24 h prior to stimulation. The most effective sequence was 5’‐CCA CTA GAG GAA ACG ACT A‐3’. Peritoneal macrophages were used for Oil Red O staining and the Dil‐ox‐LDL uptake assay (Yiyuan Biotechnology, China). RAW264.7 cell lines were used for other experiments.

### 2.3 Western blot analysis

Proteins from mice or cells were separated by 10% sodium dodecyl sulphate polyacrylamide gel electrophoresis (SDS‐PAGE) and then transferred to a polyvinylidene fluoride (PVDF) membrane for 90 min. Skim milk (5%) was used to block the PVDF membranes, and then, the membranes were incubated with primary and secondary antibodies. Finally, enhanced chemiluminescence (Millipore) was used for exposure via Amersham Imager 600.

### 2.4 ROS levels

The level of intracellular ROS was measured with the peroxide‐sensitive fluorescent probe 2’, 7’‐diacetate (Sigma‐Aldrich, Shanghai, China). The experimental group was treated with NAC (5 mmol/L) for 2 h before ox‐LDL stimulation for 24 h. RAW264.7 cell lines were exposed to DCFH‐DA (10 μmol/L) for 30 min at 37°C, and the fluorescent signal was detected via fluorescence microscopy (488 nm filter, Olympus, Tokyo, Japan).

### 2.5 Dil‐ox‐LDL uptake by macrophages

RAW264.7 cell lines were transfected with null lentivirus, IvWISP1 and shWISP1, incubated with 10 μg/mL DiI‐ox‐LDL (Yiyuan Biotechnology, China) for 2 h at 37℃, and then washed and investigated via fluorescence microscopy as described in section 2.4.

### 2.6 Oil Red O staining

Foam cells and cross‐sections of aortic sinuses were stained with Oil Red O for 30 min and then counterstained with haematoxylin. The lipid‐stained areas of slides and cross‐sections were observed and photographed using a microscope (Olympus), and the lipid droplet content was analysed using the Image‐Pro Plus image analysis software (version 6.0).

### 2.7 Plasma and supernatant analyses

Designated adipokines were quantitatively assessed in plasma and cell supernatant using the respective ELISA kits (R&D Systems GmbH) in accordance with the manufacturer's instructions. The TC (total cholesterol), LDL‐C (low‐density lipoprotein cholesterol) and HDL‐C (high‐density lipoprotein cholesterol) levels in plasma were measured by an enzyme‐labelled instrument (Molecular Devices) following the manufacturer's instructions. All mice plasma samples were isolated from blood by eyeball extirpation.

### 2.8 Immunohistochemistry

H_2_O_2_ (3%) was used to block endogenous peroxidase activity at room temperature for 10 min. The slides were rinsed thrice with PBS (phosphate‐buffered saline‐Tween) and blocked with 3% BSA (bull serum albumin) for 30 min. The samples were incubated with primary antibodies against WISP1 (1:250) and MOMA2 (1:300; Abcam, China) at 4℃ overnight, and then with a secondary antibody for 20 min at room temperature. After washing out the secondary antibody, the slides were stained with DAB for 1 min.

### 2.9 Immunofluorescence

The slides were rinsed with PBS thrice, blocked with 3% BSA for 30 min at 37℃ and then incubated with rabbit anti‐CD36 antibody (diluted 1:200) (Abcam, China) overnight at 4℃. After washing with PBS thrice, the slides were incubated with APC‐conjugated goat anti‐rabbit secondary antibody for 30 min at 37℃, washed with PBS and then counterstained with DAPI.

### 2.10 Statistical analysis

Data analyses and statistical predictions in this study were conducted through GraphPad prism 5.0 and SPSS 20.0. One‐way ANOVA was used for analysing differences among groups, and an unpaired t test was used for analysing discrepancies between groups. All experiments were independently conducted in triplicates or greater, and data have been shown as means ± standard deviation. *P* < .05 was considered statistically significant.

## 3 RESULTS

### 3.1 Basic characteristics of HFD ApoE‐/‐ mouse model

**Figure 1:**
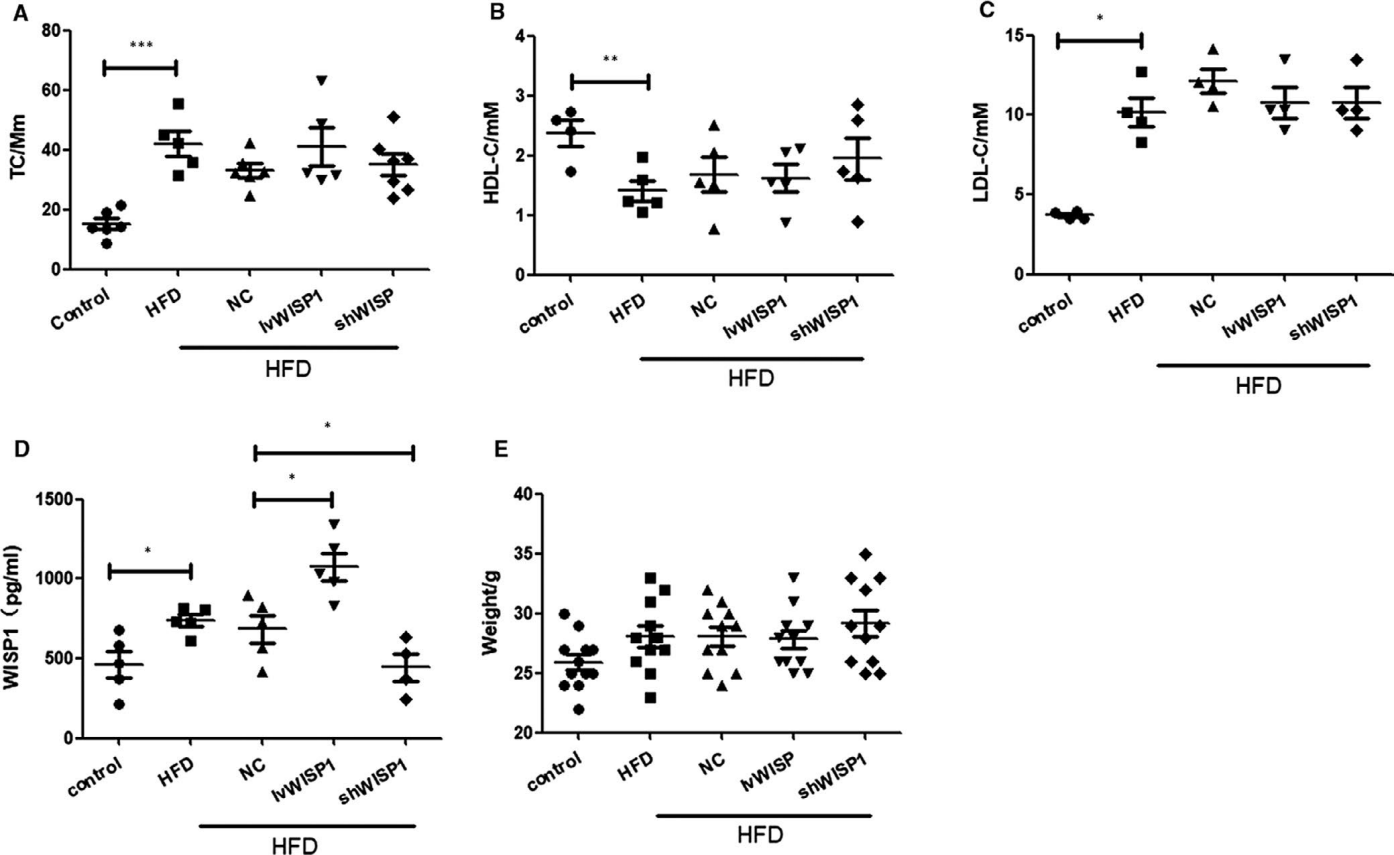
Characteristics of ApoE‐/‐ mice. TC (A, total cholesterol), HDL‐C (B, high‐density lipoprotein cholesterol), LDL‐C (C, low‐density lipoprotein cholesterol), and WISP1 (D, WNT1‐inducible signalling pathway protein 1) levels in plasma, and weight (E) were measured after 12 weeks of a high‐fat diet (*P < .05; **P < .01; data = means ±SD). HFD: high‐fat diet; NC: null lentivirus; IvWISP1: lentivirus WISP1; shWISP1: WISP1‐shRNA

After 12 weeks, the HFD (16% fat and 0.25% cholesterol) groups showed higher TC and LDL‐C levels and weight, but lower HDL‐C levels than the control group. However, WISP1 overexpression and down‐regulation had no effect on the TC, LDL‐C, HDL‐C or weight of the mice (Figure 1). Compared to the control group, HFD was found to increase the WISP1 level in serum via activation of the Wnt5a/β‐catenin pathway (Figure 4D,E,F), whereas IvWISP1 and shWISP1 significantly increased and decreased WISP1 levels in serum, respectively, compared to the HFD + NC group.

### 3.2 WISP1 affects plaque formation in ApoE‐/‐ mice

**Figure 2:**
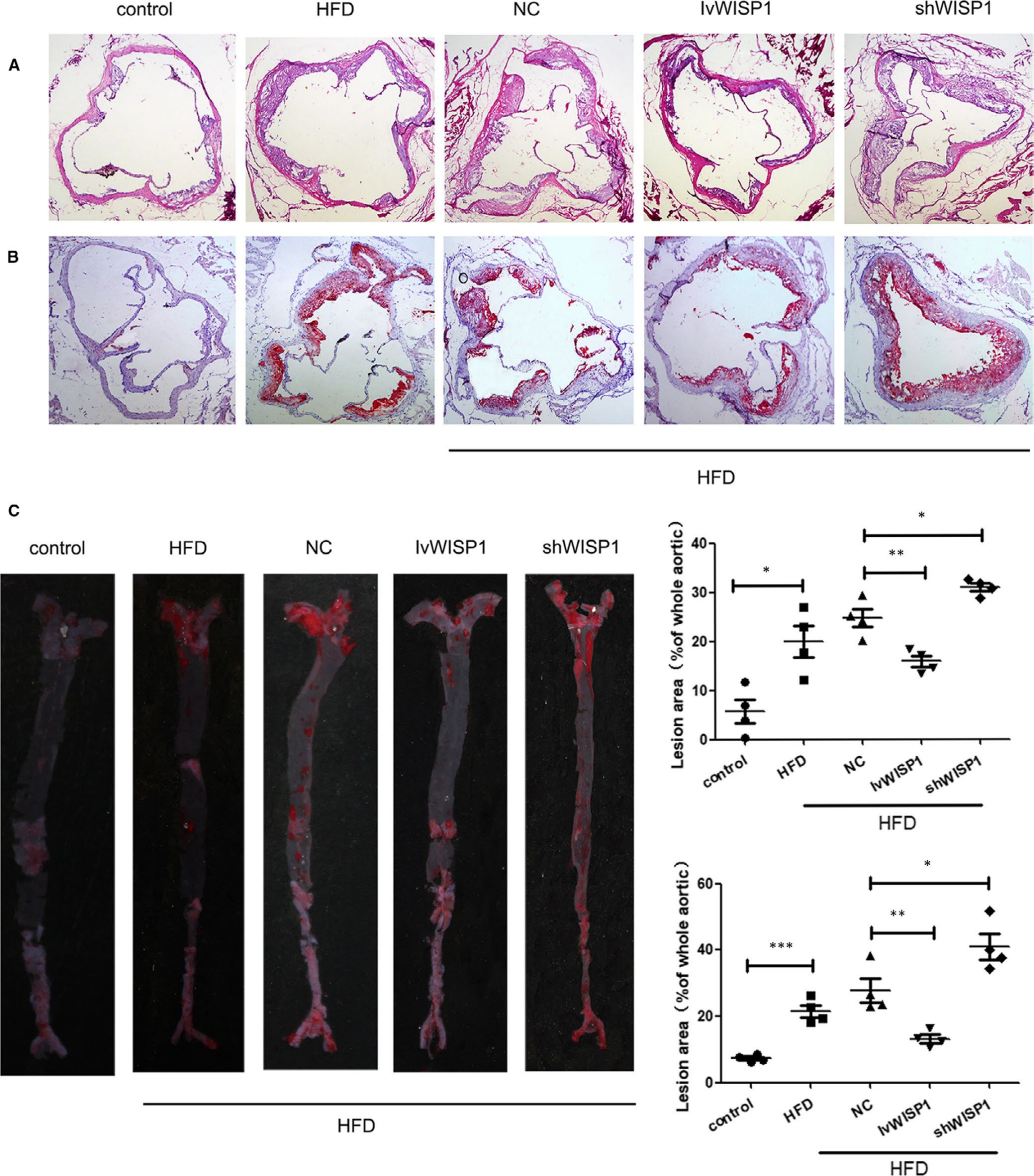
Down‐regulation of WISP1 promotes plaque formation, and WISP1 overexpression alleviates plaque formation in ApoE‐/‐ mice. (A) Representative image of haematoxylin and eosin–stained images of cross‐sections from the aortic sinus. (B) Representative image of Oil Red O staining of the aortic sinus (scale bar: 100 μm). (C) Representative image of Oil Red O staining of whole aortas (*P < .05, **P < .01, ***P < .001; data = means ±SD). HFD: high‐fat diet; NC: null lentivirus; IvWISP1: lentivirus WISP1; shWISP1: WISP1‐shRNA; WISP1: WNT1‐inducible signalling pathway protein 1

We studied images of haematoxylin and eosin (Figure 2A) and Oil Red O staining (Figure 2B) of cross‐sections from aortic sinuses. We found that IvWISP1 reduced the size of HFD‐induced atherosclerotic lesions in the aortic tissue of the mice. On the contrary, atherosclerotic lesions in the shWISP1 group were larger. The same results were observed in Oil Red O staining images of whole aortas (Figure 2C).

### 3.3 WISP1 alleviates lipid deposition and recruitment of macrophages in ApoE‐/‐ mice

**Figure 3:**
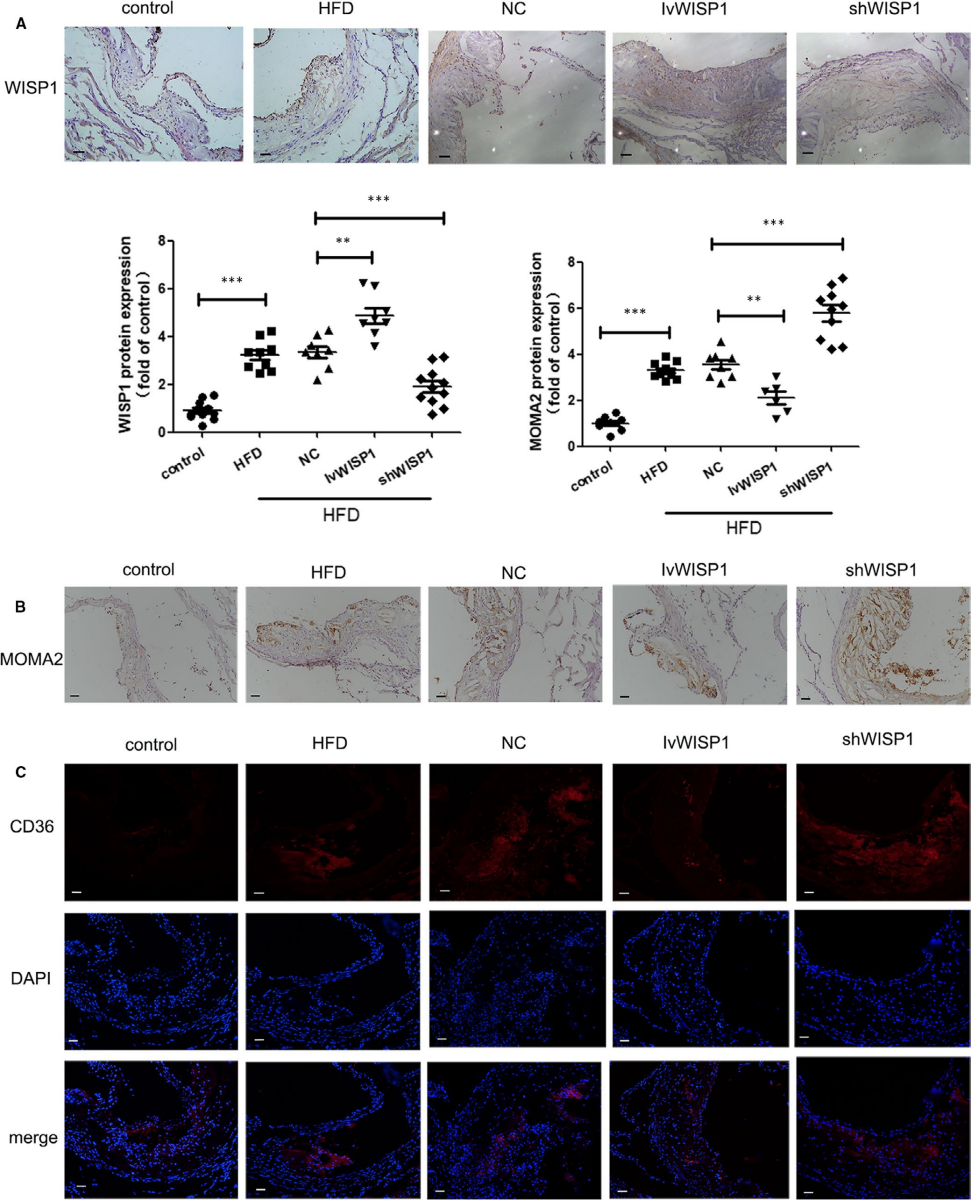
WISP1 leads to lipid deposition and recruitment of macrophages in ApoE‐/‐ mice. Immunohistochemical staining of WISP1 (A) and MOMA2 (B) from the aortic sinus (scale bar: 50 μm). (C) Immunofluorescent staining of CD36 in the aortic sinus (scale bar: 50 μm) (*P < .05, **P < .01, ***P < .001; data = means ±SD). HFD, high‐fat diet; NC, null lentivirus; IvWISP1, lentivirus WISP1; shWISP1, WISP1‐shRNA; WISP1, WNT1‐inducible signalling pathway protein 1

**Figure 4:**
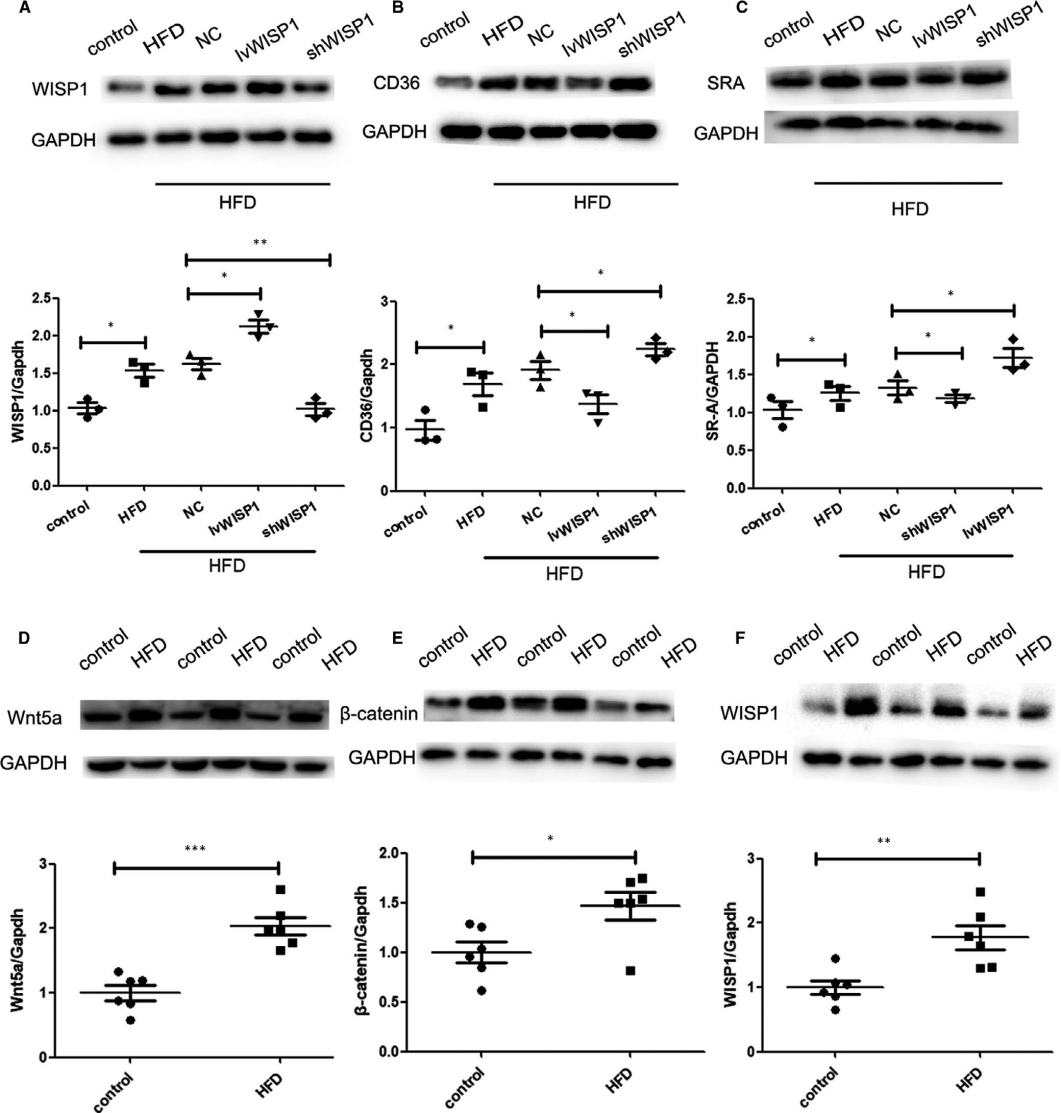
WISP1 reduces scavenger receptor (CD36 and SR‐A) levels in the arteries of ApoE‐/‐ mice. Western blot analysis of WISP1 (A), CD36 (B) and SR‐A (C). HFD can up‐regulate WISP1 via the Wnt5a/β‐catenin pathway. Western blot analysis of Wnt5a (D), β‐catenin (E) and WISP1 (F) (*P < .05, **P < .01, ***P < .001; data = means ±SD). HFD: high‐fat diet; NC: null lentivirus; IvWISP1: lentivirus WISP1; shWISP1: WISP1‐shRNA; WISP1: WNT1‐inducible signalling pathway protein

CD36 and SR‐A are important factors in lipid deposition.^30^ The expression of CD36 and SR‐A proteins increased significantly in HFD mice compared to those in the control group. Down‐regulation of WISP1 contributed to higher CD36 and SR‐A levels, while IvWISP1 mice showed decreased CD36 and SR‐A levels compared to mice in the NC group (Figure 3A,C, Figure 4A,B,C). Moreover, more macrophage recruitment in plaques was observed in HFD mice than in mice on a regular diet. Additionally, down‐regulation of WISP1 resulted in macrophage recruitment, which decreased in IvWISP1‐treated mice compared to NC group mice (*P* < .05; Figure 3B).

### 3.4 ROS mediates WISP1 expression with ox‐LDL stimulation in macrophages via Wnt5a/β‐catenin pathway

**Figure 5:**
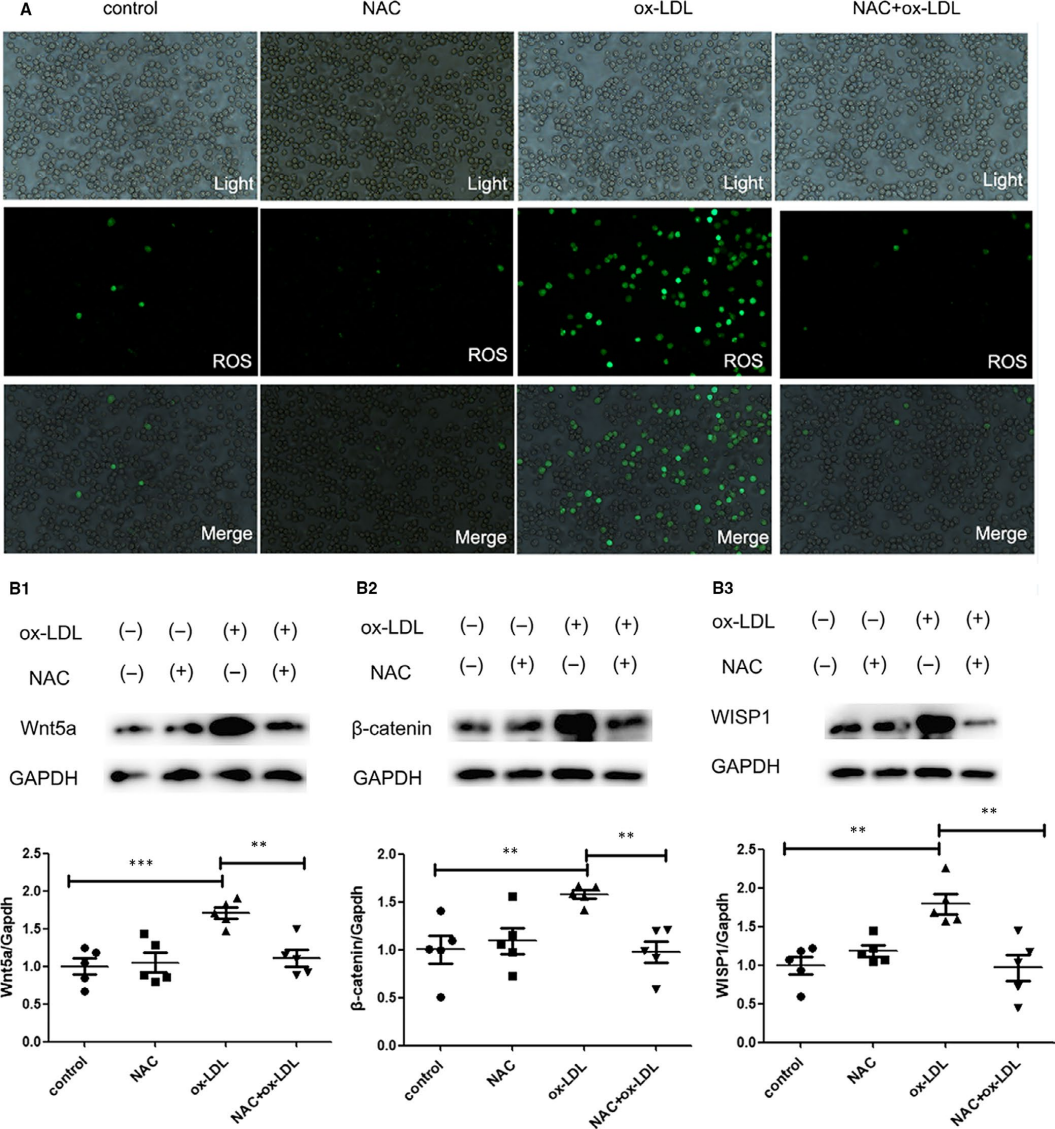
Ox‐LDL induced WISP1 expression through ROS in macrophages via the Wnt5a/β‐catenin pathway. (A) ROS levels were detected via immunofluorescence. (B) Western blot analysis of Wnt5a (B1), β‐catenin (B2) and WISP1 (B3) (*P < .05, **P < .01, ***P < .001; data = means ±SD). NAC: N‐acetylcysteine; ROS: reactive oxygen species; WISP1: WNT1‐inducible signalling pathway protein 1; ox‐LDL: oxidized low‐density lipoprotein

WISP1 protein expression apparently increased after stimulation with ox‐LDL, thereby confirming that ox‐LDL stimulation promoted WISP1 expression. As ROS production was promoted by ox‐LDL, we inspected possible mechanisms of ox‐LDL stimulation of WISP1 in macrophages. To investigate this, NAC (N‐Acetyl‐L‐cysteine), an ROS inhibitor, was used to reduce the process of ROS generation. We found that WISP1 expression in the ox‐LDL + NAC group was evidently down‐regulated compared to that in the ox‐LDL group through suppression of the Wnt5a/β‐catenin pathway. (Figure 5A,B).

### 3.5 WISP1 can alleviate lipid deposition in macrophages

**Figure 6:**
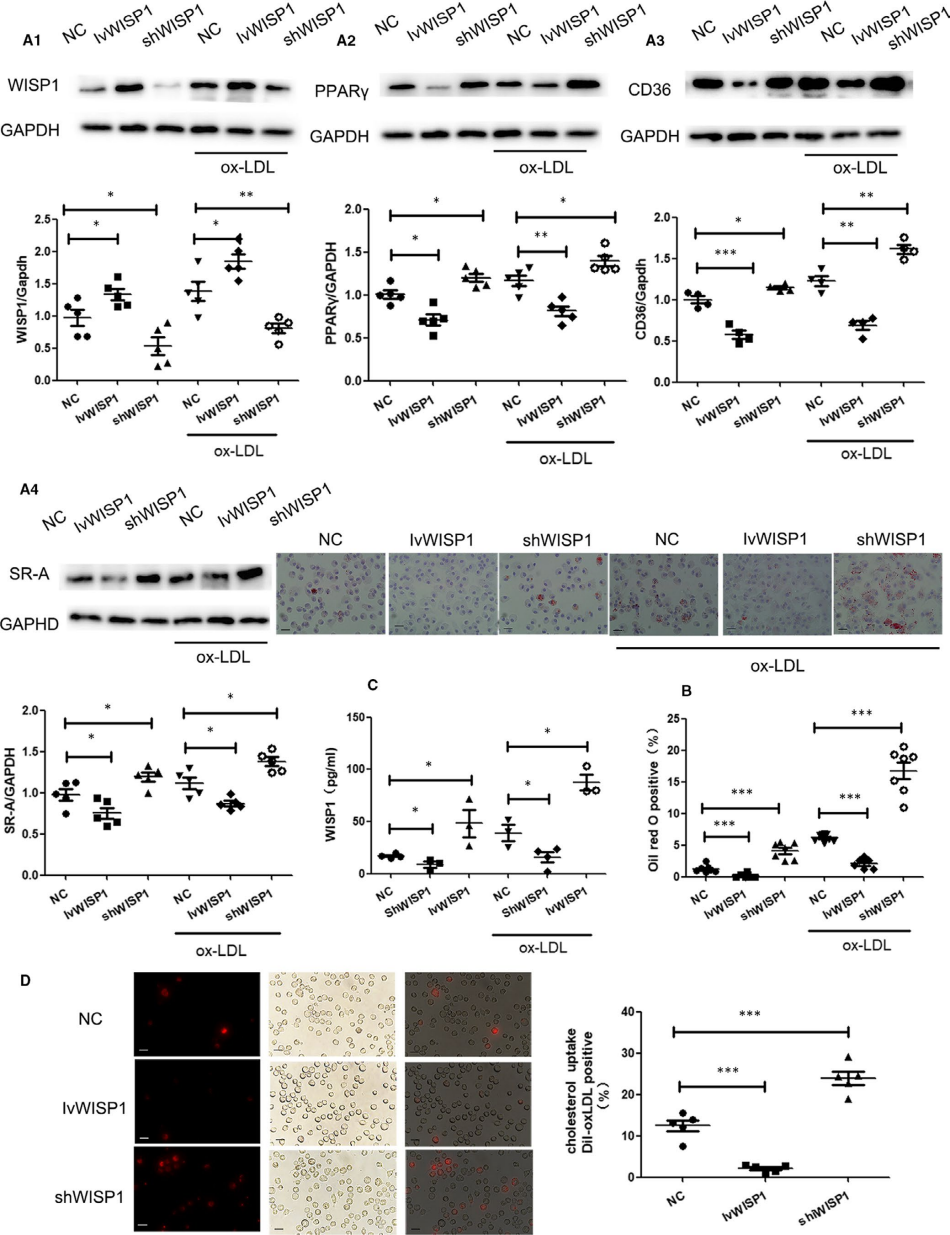
Down‐regulation of WISP1 can accelerate lipid deposition in macrophages. A: Western blot analysis of WISP1 (A1), PPARγ (A2), CD36 (A3) and SR‐A (A4) in macrophages. B: Representation Oil Red O staining of macrophage (scale bar: 25 μm, n = 7‐9). C: Wisp1 content in cell supernatant. D: Representative image of fluorescently labelled oxidized low‐density lipoprotein (Dil‐ox‐LDL) uptake in macrophages (scale bar: 25 μm, n = 5) (*P < .05, **P < .01, ***P < .001; data are means ± SD). NC: null lentivirus, IvWISP1: lentivirus WISP1, shWISP1: WISP1‐shRNA

We then determined whether WISP1 was involved in lipid deposition. After up‐regulating WISP1 expression with the help of the IvWISP1 lentivirus, the protein level of WISP1 was elevated in the cell supernatant of the macrophages (Figure 6C); this efficient up‐regulation decreased the levels of PPARγ, CD36 and SR‐A compared to the NC and NC + ox‐LDL groups, respectively. Conversely, after down‐regulating WISP1 expression with the help of the shWISP1 lentivirus, the protein levels of PPARγ, CD36 and SR‐A increased in the macrophages; this efficient inhibition also raised CD36 and SR‐A levels compared to the NC + ox‐LDL group (Figure 6A). Dil‐ox‐LDL uptake and Oil Red O staining experiments indicated that ox‐LDL could stimulate lipid deposition of macrophages. The up‐regulation of lentivector fluorescence clearly demonstrated that WISP1 could reduce the amount of lipid deposition in macrophages compared to purely the NC and ox‐LDL stimulation groups (Figure 6B,D).

### 3.6 Down‐regulation of WISP1 is involved in the activation of PPARγ and promotion of lipid deposition

**Figure 7:**
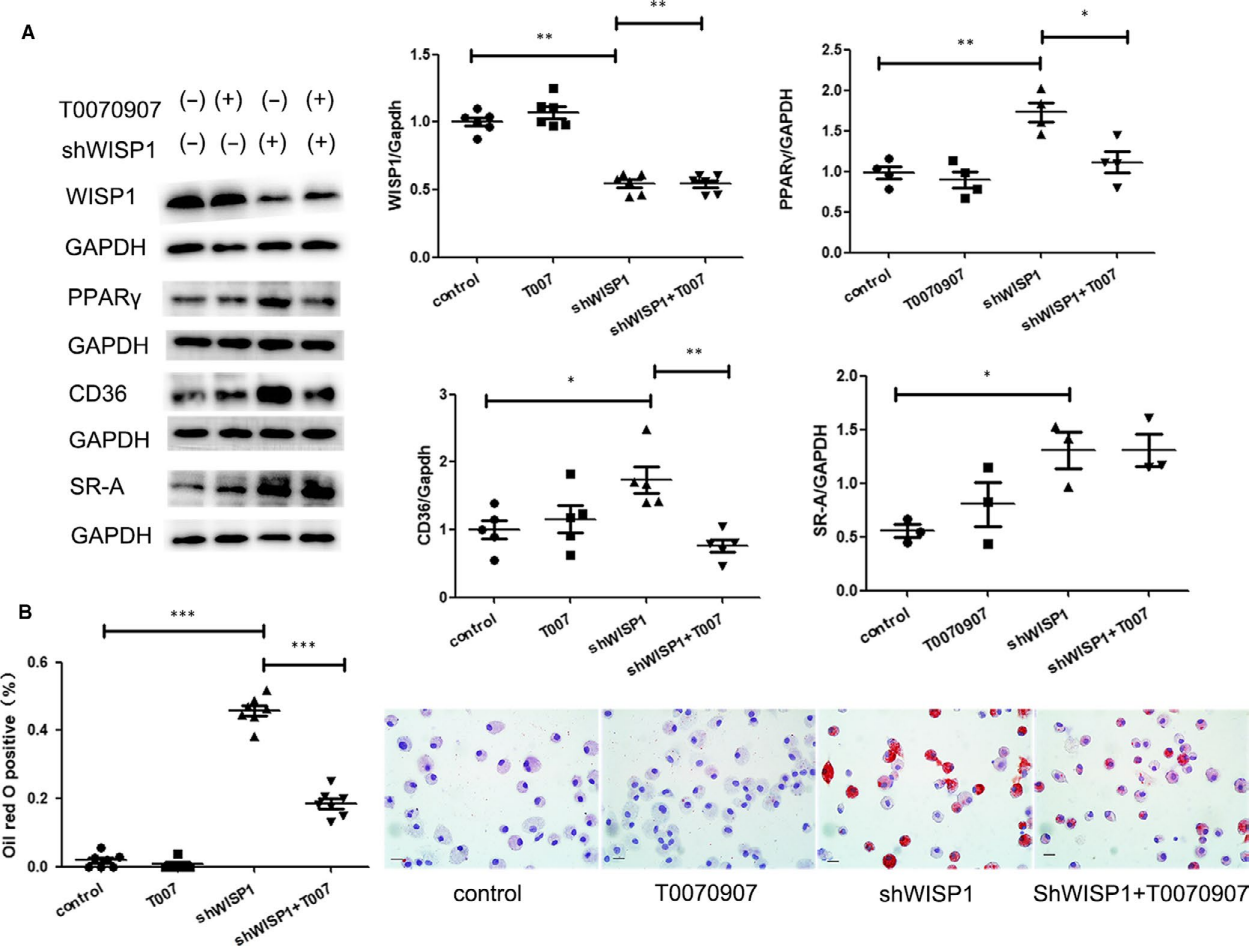
Down‐regulation of WISP1 is involved in lipid deposition through PPARγ pathway. A: Western blot analysis of WISP1, PPARγ, CD36 and SR‐A in macrophages. B: Representation of Oil Red O staining of macrophage (scale bar: 25 μm) in macrophages (*P < .05, **P < .01, ***P < .001; data are means ± SD). shWISP1: WISP1‐shRNA, T0070907: a kind of PPARγ inhibitor. WISP1‐shRNA

To analyse the function of WISP1 in the activation of PPARγ, lentivirus WISP1 and WISP1‐shRNA were used to increase and reduce WISP1 expression, respectively. Down‐regulation of WISP1 led to higher levels of PPARγ, CD36 and SR‐A compared to the NC group (*P* < .05), whereas IvWISP1 treatment had the opposite effect (*P* < .05) (Figure 7A). To further understand the mechanism, we used T0070907, an inhibitor of PPARγ, and found that it inhibited the up‐regulation of lipid deposition and CD36 expression under the condition of down‐regulated WISP1 (Figure 7B).

## 4 DISCUSSION

Atherosclerosis is known as one of the most widespread diseases, and its formation is complex, including lipid deposition, endothelial dysfunction and the propagation of these reactions.^1^ Lipid deposition is considered as a chronic inflammatory disorder.^31^ Lipid deposition in macrophage foam cells can initiate the formation of plaques and aggravate the progression of atherosclerosis. Thus, studying the mechanism of lipid deposition may provide a theoretical basis for atherosclerosis prevention.

WISP1, a member of the CCN family and a target gene of the WNT signalling pathway, is a type of adipokine. Its overexpression can be detected in visceral fat from obese individuals and can indicate insulin resistance and adipose tissue inflammation.^32^ WISP1 is also able to participate in the process of adipogenic differentiation and can participate in the immunological aspects of breast cancer progression and immune surveillance.^33^ Some studies have demonstrated that WISP1 accelerates angiogenesis in oral squamous carcinoma via VEGF‐A up‐regulation.^34^ In this study, we found that WISP1 expression was significantly elevated in high‐fat diet ApoE‐/‐ mice. Besides, we found that high‐fat diet (HFD) group could up‐regulate the expression of WISP1 via activating Wnt5a/β‐catenin pathway compared with control group in ApoE‐/‐ mice. This suggested that in atherosclerotic lesions, WISP1 was up‐regulated by activating Wnt5a/β‐catenin pathway. Interestingly, when we down‐regulated WISP1 expression, plaque formation worsened, suggesting that WISP1 played an important role in the progression of atherosclerosis. The down‐regulation of WISP1 can accelerate macrophage recruitment in plaques. Additionally, a high‐fat diet increased the weight and the levels of TC and LDL‐C in mice, but decreased HDL‐C levels. However, WISP1 inhibition and overexpression had no effect on the TC, LDL‐C and HDL‐C. Therefore, we concluded that WISP1 levels could be raised by HFD, and WISP1 can decelerate the progression of atherosclerosis.

Uncontrolled uptake of oxidized, low‐density lipoprotein (ox‐LDL) leads to the accumulation of cholesterol ester (CE), which is stored as cytoplasmic lipid droplets and subsequently triggers the formation of foam cells. The generation of foam cells is associated with the lipid homeostasis between cholesterol influx and efflux, and esterification.^35^, ^36^ The scavenger receptors (SRs) CD36 and SR class A (SR‐A) are the major receptors responsible for the uptake of ox‐LDL in macrophages.^19^ In contrast, the efflux of intracellular cholesterol to high‐density lipoprotein is mediated by reverse cholesterol transporters, including class B scavenger receptor type I (SR‐BI) and ATP‐binding cassette transporter A1 (ABCA1).^37^, ^38^, ^39^ Additionally, CD36 is highly expressed in macrophages, and its expression is regulated by multiple factors. For example, CD36 levels in peritoneal macrophages are stimulated by peroxisome proliferator‐activated receptor‐γ (PPARγ).

**Figure 8:**
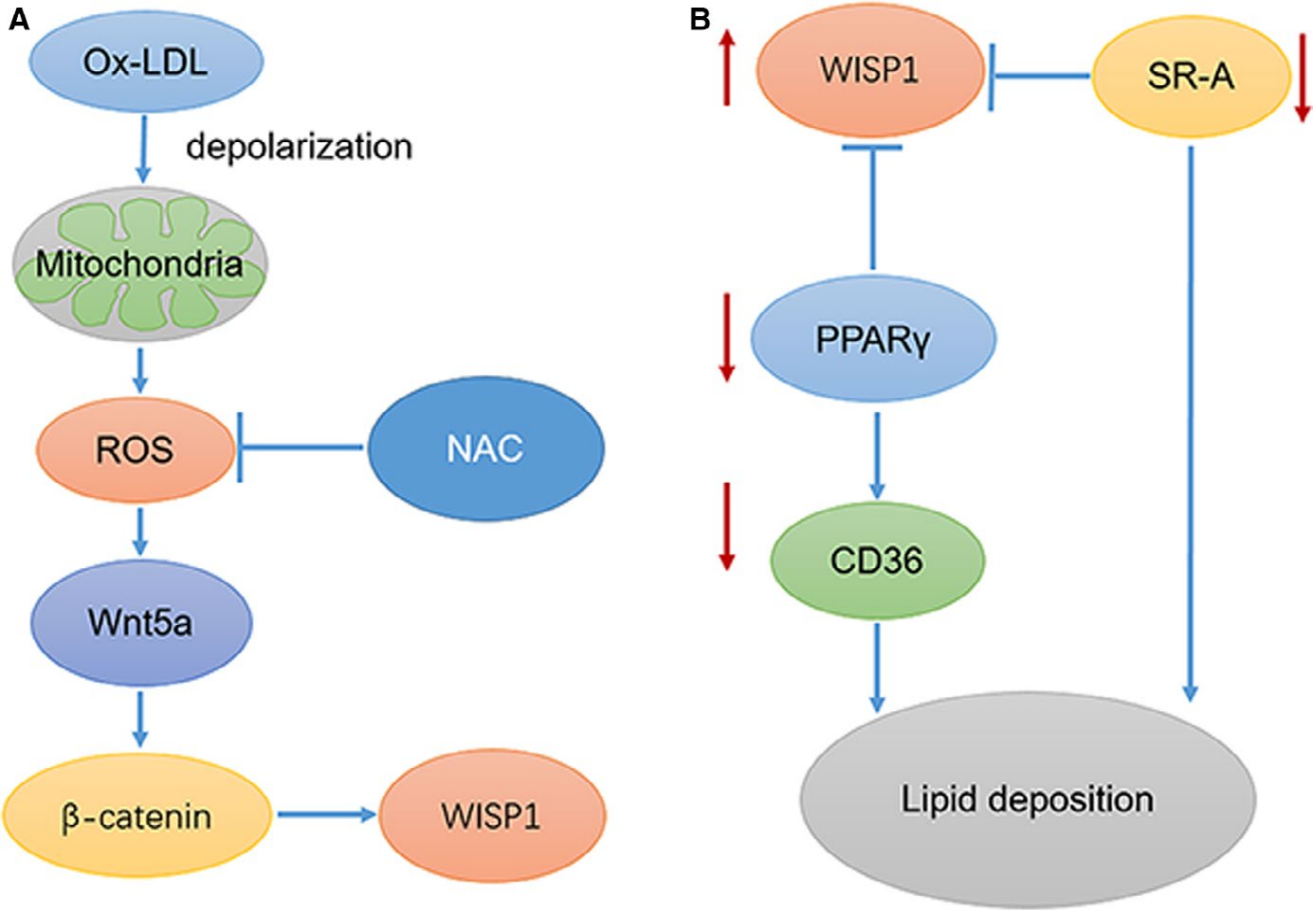
Proposed schematic model (A) Ox‐LDL can depolarize mitochondrial membrane and trigger excessive reactive oxygen species (ROS). ROS can activate Wnt5a/β‐catenin pathway and then up‐regulate expression of WISP1. While ROS inhibition NAC can reduced WISP1 expression under the stimulation of ox‐LDL. (B) WISP1 expressed in the cytosol where it catalyses ubiquitination and subsequent degradation of PPARγ through direct interaction, consequently inhibiting its transcriptional activity. Inhibition of PPARγ can inhibit its downstream CD36. Besides, WISP1 can down‐regulate the expression of SR‐A. CD36 and SR‐A are crucial for lipid deposition

PPARγ is a member of a nuclear hormone superfamily that heterodimerizes with RXR. They are transcriptional regulators of genes that can encode proteins involved in adipogenesis and lipid metabolism.^11^ Interestingly, interaction of WISP1 with PPARγ leads to proteasome‐dependent degradation of the latter, and therefore, results in the reduction of its transcriptional activity and protein level. Previous studies have shown that that PPARγ is polyubiquitinated and degraded in a proteasome‐dependent manner. Some studies have shown an additional mechanism of PPARγ regulation in which WISP1 is not only secreted, but also expressed in the cytosol where it catalyses ubiquitination and subsequent degradation of PPARγ through direct interaction, consequently inhibiting its transcriptional activity. Even in the presence of rosiglitazone, a PPARγ agonist, WISP1 continues to counteract PPARγ activation and induction of adipogenesis, suggesting that WISP1 is a new potent inhibitor of adipogenesis.^40^ Additionally, down‐regulation of WISP1 can activate the PPARγ/CD36 pathway. (Figure 8B) To investigate and further clarify the mechanism involving WISP1 and the PPARγ/CD36 pathway, we used the PPARγ inhibitor T0070907; we found it inhibited the activity of the PPARγ/CD36 pathway and lipid deposition in shWISP1‐treated macrophages. In our study, CD36 and SR‐A protein expression increased significantly in ApoE‐/‐ mice in the HFD group compared to those in the control group. Moreover, down‐regulation of WISP1 contributed to higher levels of CD36 and SR‐A, whereas IvWISP1 mice showed decreased levels of CD36 and SR‐A compared to mice in the NC group.

High levels of ox‐LDL are a primary risk factor for atherosclerosis, a common pathological condition that causes lipid deposition and foam cell formation in cardiovascular diseases. It has been proven that ox‐LDL can mediate smooth muscle apoptosis,^41^ proliferation and migration.^42^ In endothelial cells, ox‐LDL can increase pro‐inflammatory molecules, promote cell adhesion and accelerate apoptosis. Additionally, monocytes differentiate into macrophages that subsequently engulf ox‐LDL via scavenger receptors (CD36 and scavenger receptor‐A (SR‐A)), thereby leading to foam cell formation. Ox‐LDL has been shown to trigger excessive reactive oxygen species (ROS) in macrophages. Mitochondrial membrane depolarization was triggered by ox‐LDL, suggesting that ox‐LDL in macrophages took part in the disruption of mitochondrial function and triggered excessive reactive oxygen species (ROS).^43^, ^44^ Our study clearly demonstrated that WISP1 overexpression was triggered by ox‐LDL in macrophages, by activation of the Wnt5a/β‐catenin pathway (RAW264.7 cell line) (Figure 8A). Additionally, ox‐LDL treatment resulted in excessive synthesis of ROS in macrophages, but with ROS inhibition, WISP1 expression was reduced through repression of the Wnt5a/β‐catenin pathway in macrophages treated with ox‐LDL. This suggests that oxidative stress regulates WISP1 expression in macrophages treated with ox‐LDL.

Studies have shown that mtDNA damage is increased in the macrophages from atherosclerotic lesions, and this damage enhances mitochondrial oxidative stress and triggers excessive reactive oxygen species (ROS) of macrophages in atherosclerotic lesions.^45^ H2O2, as an ROS, can up‐regulate Wnt5a and its cell surface receptors, Frizzled and Ror2 in periodontal ligament cells.^46^ WNT5A is a part of the WNT family. WNT5A signalling through the Frizzled (FZD) family receptor and the LRP5/LRP6 co‐receptor transduces the canonical WNT signalling cascade for transcriptional activation of target genes of the nuclear complex, consisting of TCF/LEF, ß‐catenin, BCL9/BCL9L and PYGO1/PYGO2. WISP1 is a transcriptional target of the canonical WNT signalling pathway.^47^ Additionally, down‐regulation of WISP1 promotes Dil‐ox‐LDL uptake and Oil Red O staining accompanied by up‐regulated expression of SR‐A and CD36, which are the most important receptors for mediating phagocytosis and pinocytosis when macrophages are triggered by extracellular modified LDL. After further study, we determined that down‐regulation of WISP1 could activate the PPARγ signalling pathway and elevate CD36 levels, which are critical for lipid deposition of macrophages. Ox‐LDL can up‐regulate WISP1 with excessive reactive oxygen species (ROS) in macrophages, and this up‐regulation can serve as a kind of protection to alleviate lipid deposition by inhibiting the PPARγ/CD36 pathway in turn.

Notably, we propose that the mechanism by which WISP1 alleviates lipid deposition of atherosclerosis is by reduction of SR‐A and CD36 expression, which is crucial to lipid deposition. Also, WISP1 can inhibit the recruitment of macrophages in atherosclerosis. When macrophages were stimulated by ox‐LDL, the expression of WISP1 increased because of ROS mediation. However, up‐regulation of WISP1 could suppress foam cell formation by suppressing the PPARγ/CD36 pathway and SR‐A.

## 5 CONCLUSION

In conclusion, we demonstrated that WISP1 could alleviate lipid deposition and plaque formation in ApoE‐/‐mice. We also found that WISP1 was activated by ox‐LDL, with high levels of ROS, which played a significant role in mediating WISP1 expression. Nonetheless, down‐regulation of WISP1 can promote SR‐A and CD36 expression through the PPARγ signalling pathway. Hence, this study successfully clarified the role of WISP1 in atherosclerotic plaques, thereby providing a new therapeutic goal for atherosclerosis.

## CONFLICT OF INTEREST

The authors declare that they have no conflict of interest.

## AUTHORS' CONTRIBUTIONS

D. Liu: Induction of animal model, Western blots and cell experiment. X. Wang: Induction of animal model, Western blots and cell experiment. M. Liu: Oil Red O and other staining procedures. T. Jin: Oil Red O and other staining procedures. J. Pan: Oil Red O and other staining procedures. J. Tian: Data analysis; data interpretation. M. Gao: Manuscript revision; manuscript editing. F. An: Manuscript writing. M. All authors: Manuscript reading; approval of the final manuscript.

## Data Availability

Data are available on request from the authors.
